# Blood cholesterol in late-life and cognitive decline: a longitudinal study of the Chinese elderly

**DOI:** 10.1186/s13024-017-0167-y

**Published:** 2017-03-07

**Authors:** Chaoran Ma, Zhaoxue Yin, Pengfei Zhu, Jiesi Luo, Xiaoming Shi, Xiang Gao

**Affiliations:** 1 Department of Nutritional Sciences, The Pennsylvania State University, University Park, PA 16802 USA; 2 Division of Chronic Disease Control and Community Health, Chinese Center for Disease Control and Prevention, Beijing, 102206 China; 3 National Institute of Environmental Health, Chinese Center for Disease Control and Prevention, Beijing, 100021 China

**Keywords:** Lipid, Cognitive function, MMSE

## Abstract

**Background:**

Previous studies regarding the lipid-cognition relation in older adults are limited and have generated mixed results. We thus examined whether higher blood cholesterol concentrations were associated with faster cognitive decline in a community-based longitudinal study of Chinese elderly.

**Methods:**

The study included 1,159 Chinese adults aged over 60 years (women: 48.7%, mean age: 79.4 years), who were free of dementia, Parkinson disease and stroke at the baseline. Blood concentrations of total cholesterol (TC), high-density lipoprotein cholesterol (HDL-C), low-density lipoprotein cholesterol (LDL-C), and triglycerides (TG), were assessed at the baseline. Global cognitive functions were assessed using the Chinese Mini-Mental State Examination (MMSE) at in 2009, 2012 and 2014. Association between blood cholesterol and repeated cognitive function was analyzed with linear mixed models, adjusting for sociodemographic information, behavior and lifestyle, depression symptoms, physical examination, hypertension, and laboratory indexes.

**Results:**

Higher baseline TC and LDL-C concentrations were significantly associated with greater cognitive decline. Adjusted mean difference in cognitive decline rate, comparing two extreme quartiles, was 0.28 points (MMSE score) per year (95% confident interval (CI): -0.54,–0.02; P-trend = 0.005) for TC and 0.42 points per year (95% CI: -0.69, -0.16; P-trend = 0.006) for LDL-C. In a subgroup analysis, the associations between all lipids and cognitive decline appeared to be more pronounced among individuals aged 100 years or older (*n* = 90), relative to others.

**Conclusions:**

Higher blood concentrations of TC and LDL-C in late-life were associated with faster global cognitive decline.

**Electronic supplementary material:**

The online version of this article (doi:10.1186/s13024-017-0167-y) contains supplementary material, which is available to authorized users.

## Background

Cognitive impairment is one of the greatest causes of disability and becomes the primary public health issues globally. There are approximately 20–30% of Americans and 8% Chinese aged over 65 years with cognitive impairment, ranging from mild deficits to dementia [1–3]. Because of rapid world population ageing, the number of people living with cognitive impairment is expected to jump up dramatically. Currently, there have been no reversible treatments for cognitive impairment. Early intervention thus is significant for the prevention of cognitive impairment.

Previous studies indicated that high non-high density lipoprotein cholesterol (HDL-C) concentration in middle age was a potential risk factor for subsequent occurrence of cognitive impairment in later life [4–6]. However, the literature did not yield consistent results when it came to older adults [7–11]. In fact, the central nervous system has a separate supply and circulatory system of lipoproteins, with cholesterol solely through de novo synthesis and at a minimum from the periphery transfer [12]. On the one hand, high cholesterol is involved in synaptogenesis in the brain, facilitating compensatory repair of injured neuronal pathways in cognitive impairment [13, 14]. On the other hand, high cholesterol also plays a role in the accumulation of amyloid beta peptides, which accelerates the development of cognitive impairment [12]. However, those contradictory results in older adults may also lie in confounding factors when blood lipids and cognitive function were assessed. For example, individuals with high lipid concentrations were more likely to use of statins, which were suggested to be neuroprotective against cognitive dysfunction [15–18]. Previous studies were dominantly based on US or other high-income countries. According to the report from US Centers for Disease Control and Prevention, cholesterol-lowering medication (primarily statins) use increased with age, up to 48% of adults aged 75 and over [19]. Based on the new guidelines released by the American College of Cardiology (ACC) and the American Heart Association (AHA), a third of adults in the U.S. have been recommended to use statins, without lipid-cognition relation taken into account [20]. In this context it is of significance to understand the association between lipid profiles and cognitive function. Because only 0.4% of Chinese adults living in rural areas in China used statins, as suggested by a national survey between 2005 and 2009 [21, 22], Chinese rural areas would be an excellent setting for studying this important research question. In addition, although the risk of cognitive impairment increases with age, there has been only few studies concerning the oldest-old and there still have been no longitudinal data of a Chinese oldest old population.

In the current study, we examined whether higher blood cholesterol concentrations were associated with faster cognitive decline in a community-based longitudinal study of Chinese elderly living in rural areas.

## Methods

### Study population

This longitudinal study based on participants aged over 60 years in the Chinese Longitudinal Healthy Longevity Survey (CLHLS), an ongoing community-based study in seven longevity areas in China. CLHLS was established in half of the counties and cities in 22 of China’s 31 provinces in 1998, and then was conducted in years of 2000, 2002, 2005, 2009, 2012 and 2014 [23]. Since the wave of 2009, in-depth study has been conducted in the eight longevity areas [24], which included cognitive function, laboratory assessment, the questionnaire assessment, physical examination and anthropometric measurement.

Of 3163 potential participants aged 60 years and over, we excluded 91 people with dementia, Parkinson disease and stroke based on questionnaire, and 127 people with unreliable body mass index (BMI) (BMI < 10 or BMI > 60). We also excluded 654 participants with a Mini-Mental State Examination (MMSE) score of less than or equal to 10 because cognitive function was too impaired to be able to complete the cognitive tests or questionnaires, leaving 2,291 eligible participants. There were 1,056 participants who died during the follow-up or loss of follow-up, and 76 people with incomplete covariate information, leaving 1,159 individuals with at least two cognitive tests during the follow-up for the current analysis. Specifically, 390 participants participated cognitive test in 2009, 1,116 participants participated cognitive test in 2012, and 1072 participants participated the test in 2014. Compared with those eligible participants, individuals who died or lost to follow up, had an older age, higher percentage of women, less years of education, lower BMI, blood total cholesterol (TC), low density lipoprotein cholesterol (LDL-C) and MMSE scores at the baseline (*P* < 0.05 for all). The study was approved by the Institutional Review Board of Peking University, China. Informed consent was obtained from the participants or their representatives.

### Assessment of lipid profile

At the baseline, 12-h overnight fasting plasma TC, LDL-C, HDL-C, and triglyceride (TG) were measured by an Automatic Biochemistry Analyzer (Hitachi 7180, Japan), using commercially available diagnostic kits (Roche Diagnostic, Mannheim, Germany) in the central clinical lab at Capital Medical University in Beijing. TC and TG concentrations were both measured by standard enzymatic methods (CHOD-PAP and GPO-PAP; Roche Diagnostics). HDL-C was measured by a direct method, and LDL-C was determined by the Friedewald formula (LDL-C = TC - HDL-C - TG/2.17 (in mmol/L)). LDL-C plays a role in forming plaque, a thick, hard deposit that can clog arteries and make them less flexible, i.e., atherosclerosis. Less than 3.37 mmol/L of LDL-C concentration is considered normal. TG is used to store excess energy as another type of fate and high levels of TG are associated with atherosclerosis. The normal range of TG concentration is less than 1.70 mmol/L. HDL-C helps remove LDL-C from the arteries, and higher than 1.55 mmol/L of HDL-C concentration is the normal expected level. As a combination of LDL-C, TG and HDL-C, high concentrations of TC is also an atherogenic marker. The desirable concentration of TC is less than 5.18 mmol/L.

### Assessment of cognitive function

Global cognitive functions were assessed at 2009, 2012 and 2014, using the validated Chinese version of MMSE, which was culturally translated into Chinese language on the basis of the international standard of MMSE questionnaire [25]. MMSE scores ranged from 0 to 30, with the indication that higher scores reflect better cognitive function.

### Assessment of potential covariates

Potential covariates included sociodemographic information (age, sex, and education levels), behavior and lifestyle (smoking status, alcohol intake, and physical activity), depression symptoms, physical examination (BMI, and waist circumference), hypertension, and laboratory indexes (plasma glucose, C-reactive protein and uric acid). Among the above data, sociodemographic information, behavior and lifestyle and depression symptoms were collected at baseline via questionnaires, Weight and height were measured by trained medical personnel. BMI was calculated as body weight (kg) divided by the square of height (m^2^). The waist circumference was measured to the nearest 0.1 cm at the midpoint between the bottom of the rib cage and the top of the iliac crest at the end of exhalation. Systolic and diastolic blood pressures were measured twice from the seated position using a mercury sphygmomanometer. The average of the two readings was used for analysis. Plasma glucose and uric acid were assessed together with the lipid profile by an Automatic Biochemistry Analyzer (Hitachi 7180, Japan), using commercially available diagnostic kits (Roche Diagnostic, Mannheim, Germany).

### Statistical analyses

To evaluate the relation of blood lipid profile to cognition we examined mean performance at each assessment using linear mixed models (which permits examination of each time point, taking into account lipid profile status at baseline and an interaction between lipid profile status and time). We considered a number of potential confounders for inclusion in each model: model 1 was adjusted for sociodemographic information; model 2 was adjusted for sociodemographic information, behavior and lifestyle; model 3 was adjusted for all the covariates stated before. Education was categorized as illiteracy, 1–6 years or ≥6 years. Smoking status was categorized as non-smoker and smoker (0, 0.7–20.4, 20.5–44.4, or 44.5–220 pack-year). Alcohol intake was categorized as non-drinker and drinker (0, 0.4–2.11, 2.12–4.67, or 4.68–67.7 servings/d). BMI was categorized by 17.5, 17.5–23.0, 23.0–27.9, or ≥28.0 kg/m^2^. Waist circumference was categorized by 50–73, 74–80, 81–88, or 89–155 cm. Plasma glucose was categorized by 0.15–3.93, 3.94–4.68, 4.69–5.41, or 5.42–36.04 mmol/L. C-reactive protein was categorized by <1, 1–2.9, or ≥3 mg/L. Uric acid was categorized by <240, 240–360, or ≥360 for women, and <240, 240–420, or ≥420 μmol/L for men. Physical activity, depression symptoms and hypertension were classified as yes or no. The estimate for the effect of “lipid profile status” reflected the cross-sectional impact of the lipid profile status on cognitive function at baseline and was presented as the “cross-sectional effect.” The estimate for the effect of “time” reflected the annual change in cognitive function and was presented as “change over time.” The estimate for “interaction” of the lipid profile status and time reflected the additional annual impact of the lipid profile status on cognitive function and was presented as “additional annual effect.” We examined linear trends across quartiles of a given lipid using a continuous variable in which participants in a given category were assigned the median value.

Given the possibility that age (60–79 y, 80–99 y, and 100+ y) and sex at baseline could modify the association between lipid profile and cognitive decline, interaction terms for these variables and lipid profile were evaluated, and subgroup analyses by these variables were further conducted. All statistical analyses were conducted using SAS version 9.4 (SAS Institute, Inc, Cary, NC). Formal hypothesis testing will be 2-sided with a significant level of 0.05.

## Results

Of 1,159 participants, 48.7% were women. The mean age was 79.4 years (ranged 60–112 y) and the mean education years was two years (ranged 0–19 years). Compared with those with a lower TC concentration, participants with a higher TC concentration had an older age, a higher percentage of women and hypertension, a higher BMI, blood uric acid and LDL-C concentration, and a larger waist circumference (*P* < 0.05) (Table 1). No differences were observed with other covariates.

**Table 1:** Characteristics by quartile of baseline total cholesterol^a^

	Quartile 1	Quartile 2	Quartile 3	Quartile 4	P trend^b^
	0.35–3.46 mmol/L	3.47–4.14 mmol/L	4.15–4.84 mmol/L	4.85–8.79 mmol/L	
No.	290	287	293	289	
Age, y	78.4 (0.64)	79.0 (0.63)	80.4 (0.64)	80.0 (0.64)	0.03
Women, %	39.3	37.3	56.7	61.3	<0.001
Education, y	2.34 (0.18)	2.46 (0.18)	2.29 (0.18)	2.81 (0.19)	0.14
Smoking status, pack-year	8.59 (1.12)	8.87 (1.14)	7.59 (1.11)	10.98 (1.13)	0.25
Alcohol intake, servings/d	0.98 (0.47)	1.99 (0.48)	1.83 (0.47)	1.58 (0.48)	0.45
Physical activity, %	21.1	19.5	20.1	23.7	0.84
Depression symptoms, %	7.0	6.9	5.6	6.9	0.23
Body mass index, kg/m^2^	21.0 (0.22)	21.6 (0.22)	21.6 (0.22)	22.5 (0.22)	<0.001
Waist circumference, cm	80.2 (0.63)	79.6 (0.64)	81.4 (0.63)	83.5 (0.63)	<0.001
Hypertension, %	49.7	55.4	61.4	59.2	0.02
LDL-C, mmol/L	1.66 (0.03)	2.11 (0.03)	2.59 (0.03)	3.45 (0.03)	<0.001
Glucose, mmol/L	5.10 (0.13)	4.71 (0.13)	4.99 (0.13)	4.88 (0.13)	0.51
Uric acids, μmol/L	272.5 (4.90)	278.2 (4.94)	284.1 (4.87)	299.9 (4.94)	<0.001

^a^Values are mean (standard error) adjusted for age and sex

^b^Adjusted for age and sex

Higher concentrations of TC and LDL-C were associated with faster decline in the MMSE score over time (Table 2). Adjusted mean difference in annual cognitive decline rate, comparing two extreme quartiles, was -0.28 points (95% confidence interval (CI): -0.54, -0.02; P trend = 0.005) for TC and -0.42 points (95% CI: -0.69, -0.16; P trend = 0.006) for LDL-C. The magnitudes were equivalent to 2.0- and 3.0-year ageing observed in the current study, respectively. In contrast, we did not observe significant relations between HDL-C/TG concentrations and cognitive decline (P trend > 0.05 for both).

**Table 2:** Mean difference and 95% confidence interval in annual cognitive decline according to quartile of lipid concentrations

	Quartile 1	Quartile 2	Quartile 3	Quartile 4	Cognitive decline for each mmol/L increment	P Trend
TC						
	0.35–3.46 mmol/L	3.47–4.14 mmol/L	4.15–4.84 mmol/L	4.85–8.79 mmol/L		
N	290	287	293	289		
Median (mmol/L)	2.88	3.83	4.45	5.38		
Model 1^a^	0 (ref.)	−0.10 (−0.33, 0.13) ))	−0.25 (−0.49, −0.01)	−0.26 (−0.52, −0.003)	−0.09 (−0.16, −0.02)	0.009
Model 2^b^	0 (ref.)	−0.10 (−0.33, 0.13)	−0.26 (−0.50, −0.02)	−0.27 (−0.52, −0.01)	−0.09 (−0.16, −0.02)	0.008
Model 3^c^	0 (ref.)	−0.12 (−0.36, 0.12)	−0.28 (−0.52, −0.03)	−0.28 (−0.54, −0.02)	−0.10 (−0.17, −0.03)	0.005
LDL-C						
	0.20–1.85 mmol/L	1.86–2.39 mmol/L	2.40–2.97 mmol/L	2.98–5.69 mmol/L		
N	290	287	291	291		
Median (mmol/L)	1.51	2.14	2.66	3.44		
Model 1^a^	0 (ref.)	−0.24 (−0.47, −0.02)	−0.18 (−0.43, 0.06)	−0.38 (−0.64, −0.12)	−0.14 (−0.25, −0.03)	0.01
Model 2^b^	0 (ref.)	−0.25 (−0.47, −0.02)	−0.19 (−0.43, 0.06)	−0.40 (−0.66, −0.14)	−0.15 (−0.26, −0.04)	0.008
Model 3^c^	0 (ref.)	−0.26 (−0.48, −0.03)	−0.20 (−0.45, 0.04)	−0.42 (−0.69, −0.16)	−0.16 (−0.27, −0.04)	0.006
HDL-C						
	1.48–3.18 mmol/L	1.22–1.47 mmol/L	1.01–1.21 mmol/L	0.01–1.00 mmol/L		
N	293	286	289	291		
Median (mmol/L)	0.88	1.12	1.32	1.70		
Model 1^a^	0 (ref.)	0.12 (−0.11, 0.35)	−0.02 (−0.26, 0.22)	−0.04 (−0.30, 0.21)	0.01 (−0.24, 0.27)	0.91
Model 2^b^	0 (ref.)	0.12 (−0.12, 0.35) −0.03 (−0.27, 0.21)9-0.11, 0.36)	−0.03 (−0.29, 0.22) 9–0.27	−0.04 (−0.20, 0.22) 99(( 9–0.3	0.02 (−0.23, 0.28)	0.87
Model 3^c^	0 (ref.)	0.10 (−0.14, 0.34)	−0.04 (−0.29, 0.21)	−0.05 (−0.31, 0.21)	0.01 (−0.25, 0.27)	0.93
TG						
	0.13–0.67 mmol/L	0.68–0.92 mmol/L	0.93–1.32 mmol/L	1.33–8.05 mmol/L		
N	287	296	287	289		
Median (mmol/L)	0.54	0.79	1.09	1.89		
Model 1^a^	0 (ref.)	0.17 (−0.13, 0.46)	0.14 (−0.15, 0.44)	0.16 (−0.13, 0.45)	0.06 (−0.02, 0.14)	0.13
Model 2^b^	0 (ref.)	0.17 (−0.12, 0.47)	0.14 (−0.15, 0.44)	0.16 (−0.12, 0.45)	0.06 (−0.02, 0.14)	0.13
Model 3^c^	0 (ref.)	0.17 (−0.13, 0.47)	0.14 (−0.16, 0.44)	0.18 (−0.11, 0.47)	0.06 (−0.02, 0.14)	0.12

*Abbreviation*: *TC* total cholesterol, *LDL-C* low density lipoprotein cholesterol, *HDL-C* high density lipoprotein cholesterol, *TG* triglyceride

^a^Adjusted for age, sex and education (illiteracy, 1–6 years, or ≥6 years)

^b^Adjusted for age, sex, education, smoking status (non-smoker and smoker (0.7–20.4, 20.5–44.4, or 44.5–220 pack-year)), alcohol intake (non-drinker and drinker (0.4–2.11, 2.12–4.67, or 4.68–67.7 servings/d)), physical activities (yes/no), depression symptoms (yes/no)

^c^Adjusted for age, sex, education, smoking status, alcohol intake, physical activity, depression, BMI (<17.5, 17.5–23.0, 23.0–27.9, or ≥28.0 kg/m^2^), waist circumference (50–73, 74–80, 81–88, or 89–155 cm), hypertension (yes/no), plasma glucose (0.15–3.93, 3.94–4.68, 4.69–5.41, or 5.42–36.04 mmol/L), C-reactive protein (<1, 1–2.9, or ≥3 mg/L) and uric acid (women: <240, 240–360, or ≥360; men: <240, 240–420, or ≥420 μmol/L)

**Fig. 1:**
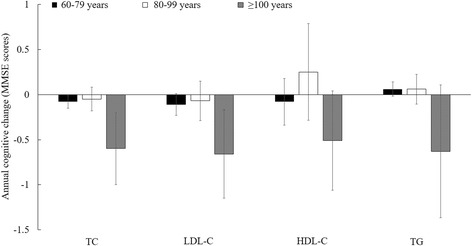
Mean difference in annual cognitive change for each mmol/L increment of lipid concentrations, stratified by age ^a^. Abbreviation: TC = total cholesterol; LDL-C = low density lipoprotein cholesterol; HDL-C = high density lipoprotein cholesterol; TG = triglyceride. P interaction > 0.50 for all, suggesting that the association between lipids and cognitive decline was not modified by age. ^a^Adjusted for age, sex, education (illiteracy, 1–6 years, or ≥6 years), smoking status (non-smoker and smoker (0.7–20.4, 20.5–44.4, or 44.5–220 pack-year)), alcohol intake (non-drinker and drinker (0.4–2.11, 2.12–4.67, or 4.68–67.7 servings/d)), physical activities (yes/no), depression symptoms (yes/no), BMI (<17.5, 17.5–23.0, 23.0–27.9, or ≥28.0 kg/m^2^), waist circumference (50–73, 74–80, 81–88, or 89–155 cm), hypertension (yes/no), plasma glucose (0.15–3.93, 3.94–4.68, 4.69–5.41, or 5.42–36.04 mmol/L), C-reactive protein (<1, 1–2.9, or ≥3 mg/L) and uric acid (women: <240, 240–360, or ≥360; men: <240, 240–420, or ≥420 μmol/L)

**Fig. 2:**
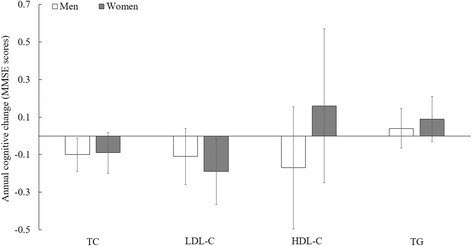
Mean difference in annual cognitive change for each mmol/L increment of lipid concentrations, stratified by sex ^a^. Abbreviation: TC = total cholesterol; LDL-C = low density lipoprotein cholesterol; HDL-C = high density lipoprotein cholesterol; TG = triglyceride.P interaction > 0.50 for all, suggesting that the association between lipids and cognitive decline was not modified by sex. ^a^ Adjusted for age, sex, education (illiteracy, 1–6 years, or ≥6 years), smoking status (non-smoker and smoker (0.7–20.4, 20.5–44.4, or 44.5–220 pack-year)), alcohol intake (non-drinker and drinker (0.4–2.11, 2.12–4.67, or 4.68–67.7 servings/d)), physical activities (yes/no), depression symptoms (yes/no), BMI (<17.5, 17.5–23.0, 23.0–27.9, or ≥28.0 kg/m^2^), waist circumference (50–73, 74–80, 81–88, or 89–155 cm), hypertension (yes/no), plasma glucose (0.15–3.93, 3.94–4.68, 4.69–5.41, or 5.42–36.04 mmol/L), C-reactive protein (<1, 1–2.9, or ≥3 mg/L) and uric acid (women: <240, 240–360, or ≥360; men: <240, 240–420, or ≥420 μmol/L)

We did not find any significant interaction between age, sex, and cognitive decline (P for interaction >0.05 for all; Figs. 1 and 2). However, in a subgroup analysis, the associations between all lipids and cognitive decline appeared to be more pronounced among individuals aged 100 years or older (*n* = 90), relative to others (Fig. 1 & Additional file 1).

## Discussion

In this community-based longitudinal study of over 1,100 Chinese older adults living in rural area, we observed that higher blood concentrations of TC and LDL-C were associated with faster cognitive decline, independent of numerous potential co-determinants of decline, including diabetes, hypertension, and blood uric acids concentration.

Consistent with our findings, one 7-year longitudinal study with 6,855 French participants (mean age = 74 y) showed that higher TC and LDL-C concentrations in men were associated with a higher risk of cognitive decline over time [7]. Similar results were observed in another longitudinal study of 1037 US postmenopausal women with 4 years of follow-up (mean age = 71 y), reporting that individuals with decrement of TC over time was associated with lower odds of impairment [8]. In contrast, there have been a few US-based studies reporting that total serum cholesterol concentrations in subjects older than 65 years were not associated with the incidence of dementia during follow-up [10, 11]. Given that participants in those longitudinal studies were all Caucasians, racial and ethnic differences might account for the result discrepancies. Further, the cholesterol-lowering medication (primarily statins) use was up to 66.5% in high-income countries [22], while the using rate in China was only 1.7% throughout the country, much less in the rural areas (0.4%) [21, 22]. Consistently, in another national survey in China based on 23,129 Chinese adults aged 30-79 years with cardiovascular disease, only 1.4% of participants used statins during 2004–08; the prevalence of statin use was significantly lower among those with lower education and household income, relative to their counterparts [26]. In this context, we speculate that the different results observed in our studies and some previous studied conducted in high-income counties could be partially explained by statin use. However, there have been no longitudinal studies, to our knowledge, on the lipid-cognition relation in general population, stratified by statin use.

We also found that the lipid-cognition association was more pronounced in individuals aged 100 years or older, relative to others. This could be due to the faster cognitive decline rate in this age group, relative to their younger counterparts. However, significant association between lipid and cognitive function was not observed in a previous cross-sectional study conducted in centenarians [27]. One possible explanation for this discrepancy may be the current study’s longer follow up period, which may have better captured cognitive trajectories. It is also possible that cardiovascular events due to the low prevalence of statin use could lead to cognitive decline. However, the interaction between age and cholesterol concentrations was not significant in the current study, which was probably due to the small number in centenarian group (*n* = 90). We cannot exclude the possibility of chance finding.

Some possible biological mechanisms may explain the observed association between high cholesterol concentrations and faster cognitive decline in the current study. Compared to cholesterol of the periphery with de novo synthesis and provided by diet half and half, brain cholesterol is stable, with the major input solely through de novo synthesis locally rather than transfer from the periphery. Therefore, the central nervous system contains its own supple of lipoproteins [12]. Brain cholesterol has been implicated to play a role in altering the degradation of the amyloid precursor protein, which could have impact on the accumulation of amyloid beta peptides, contributing to the pathogenesis of dementia [12]. In addition, hypercholesterolemia is involved in atherogenesis, which could result in macro- and micro- vascular diseases, deteriorating cognitive function both at subclinical [28, 29] and clinical levels [30].

Strengths of this study include its longitudinal design, inclusion of centenarians, and incorporation of time-dependent covariates. However, our study was limited by lack of a comprehensive neuropsychological battery to capture all detailed aspects of cognitive function. The MMSE is a relatively simple screening tool for dementia. Nevertheless, we measured the MMSE at three time points, which would allow us to capture the global pattern of cognitive change over time. Residual confounding is of concern because we did not collect information on ApoE genotype and dietary intake. Another limitation is high rate of loss to follow-up due to the old age and low education status of our participants. The diagnoses of dementia, Parkinson disease and stroke were not confirmed by review of medical records.

## Conclusions

In conclusion, higher blood concentrations of TC and LDL-C in late-life were associated with faster global cognitive decline in this older Chinese rural population. The associations appeared to be stronger among those aged 100 years or older. Further studies with ApoE data available, larger sample size, and a comprehensive neuropsychological battery to capture specific cognitive effects are warranted to confirm our findings.

## Additional file

Additional file 1: Mean difference in annual cognitive change for each mmol/L increment of lipid concentrations, stratified by age. (DOCX 13 kb)

## Abbreviations

ACC: American College of Cardiology
AHA: American heart association
BMI: Body mass index
CLHLS: Chinese longitudinal healthy longevity survey
HDL-C: High-density lipoprotein cholesterol
LDL-C: Low-density lipoprotein cholesterol
MMSE: Mini-mental state examination
TC: Total cholesterol
TG: Triglyceride.
