# Supplementary material for: Blood cholesterol in late-life and cognitive decline: a longitudinal study of the Chinese elderly
Source: Mol Neurodegener. 2017 Mar 7;12:24. doi: 10.1186/s13024-017-0167-y (PMC5341475; doi:10.1186/s13024-017-0167-y)
Supplement: Additional file 1: — Mean difference in annual cognitive change for each mmol/L increment of lipid concentrations, stratified by age. (DOCX 13 kb) [file 13024_2017_167_MOESM1_ESM.docx]

Mean difference in annual cognitive change for each mmol/L increment of lipid concentrations, stratified by age ^a^

|  | 60-79 years | 80-99 years | ≥100 years | P interaction |
| --- | --- | --- | --- | --- |
| N | 607 | 462 | 90 |  |
| TC | -0.08 (-0.15, -0.004) | -0.05 (-0.18, 0.08) | -0.6 (-1.02, -0.18) | 0.27 |
| LDL-C | -0.09 (-0.21, 0.03) | -0.06 (-0.27, 0.16) | -0.66 (-1.16, -0.15) | 0.28 |
| HDL-C | -0.07 (-0.34, 0.19) | 0.24 (-0.29, 0.77) | -0.48 (-1.92, 0.95) | 0.47 |
| TG | 0.06 (-0.02, 0.14) | 0.07 (-0.09, 0.24) | -0.63 (-1.37. 0.10) | 0.38 |

Abbreviation: TC = total cholesterol; LDL-C = low density lipoprotein cholesterol; HDL-C = high density lipoprotein cholesterol; TG = triglyceride.

P interaction > 0.50 for all, suggesting that the association between lipids and cognitive decline was not modified by age.

^a^ Adjusted for age, sex, education (illiteracy, 1-6 years, or ≥6 years), smoking status (non-smoker and smoker (0.7-20.4, 20.5-44.4, or 44.5-220 pack-year)), alcohol intake (non-drinker and drinker (0.4-2.11, 2.12-4.67, or 4.68-67.7 servings/d)), physical activities (yes/no), depression symptoms (yes/no), BMI (<17.5, 17.5-23.0, 23.0-27.9, or ≥28.0 kg/m^2^), waist circumference (50-73, 74-80, 81-88, or 89-155 cm), hypertension (yes/no), plasma glucose (0.15-3.93, 3.94-4.68, 4.69-5.41, or 5.42-36.04 mmol/L), C-reactive protein (<1, 1-2.9, or ≥3 mg/L) and uric acid (women: <240, 240-360, or ≥360; men: <240, 240-420, or ≥420 μmol/L).
